# Convolutional neural networks to predict brain tumor grades and Alzheimer’s disease with MR spectroscopic imaging data

**DOI:** 10.1371/journal.pone.0268881

**Published:** 2022-08-24

**Authors:** Jacopo Acquarelli, Twan van Laarhoven, Geert J. Postma, Jeroen J. Jansen, Anne Rijpma, Sjaak van Asten, Arend Heerschap, Lutgarde M. C. Buydens, Elena Marchiori

**Affiliations:** 1 Radboud University Nijmegen, Institute for Computing and Information Science, Nijmegen, The Netherlands; 2 Radboud University Nijmegen, Institute for Molecules and Materials, Nijmegen, The Netherlands; 3 Department of Radiology and Nuclear Medicine, Radboud University Medical Center Nijmegen, Nijmegen, The Netherlands; University of Oklahoma, UNITED STATES

## Abstract

**Purpose:**

To evaluate the value of convolutional neural network (CNN) in the diagnosis of human brain tumor or Alzheimer’s disease by MR spectroscopic imaging (MRSI) and to compare its Matthews correlation coefficient (MCC) score against that of other machine learning methods and previous evaluation of the same data. We address two challenges: 1) limited number of cases in MRSI datasets and 2) interpretability of results in the form of relevant spectral regions.

**Methods:**

A shallow CNN with only one hidden layer and an ad-hoc loss function was constructed involving two branches for processing spectral and image features of a brain voxel respectively. Each branch consists of a single convolutional hidden layer. The output of the two convolutional layers is merged and fed to a classification layer that outputs class predictions for the given brain voxel.

**Results:**

Our CNN method separated glioma grades 3 and 4 and identified Alzheimer’s disease patients using MRSI and complementary MRI data with high MCC score (Area Under the Curve were 0.87 and 0.91 respectively). The results demonstrated superior effectiveness over other popular methods as Partial Least Squares or Support Vector Machines. Also, our method automatically identified the spectral regions most important in the diagnosis process and we show that these are in good agreement with existing biomarkers from the literature.

**Conclusion:**

Shallow CNNs models integrating image and spectral features improved quantitative and exploration and diagnosis of brain diseases for research and clinical purposes. Software is available at https://bitbucket.org/TeslaH2O/cnn_mrsi.

## Introduction

Brain diseases are commonly diagnosed by Magnetic resonance imaging (MRI) to identify anatomical deviations, and in selected cases by MR spectroscopy (MRS) to assess metabolic abnormalities [1, 2]. MR spectroscopic imaging (MRSI) provides metabolic information in spectra from voxels in a 2 or 3D grid overlaying the brain. MRS can aid in the determination of type and severity of brain diseases and in the case of local or heterogeneous lesions to classify voxels into healthy versus non-healthy or degrees of disease severity, such as in brain tumors [3–12].

In the diagnosis of brain diseases by MRI, proper image segmentation is important to visualize anatomical structures and to identify pathological areas. Instead, in the diagnosis by MRSI the focus has been more on feature selection algorithms to identify metabolic abnormalities [13, 14]. In most MRSI studies, MRI is used for anatomical guidance, but it has been demonstrated that combining it with information of MRI’s improves diagnosis [4, 10, 13, 15–18].

Convolutional neural networks (CNNs) [19] are successful in medical image segmentation [20–23]. CNNs are a type of artificial neural network [24] and are the current state-of-art for image classification [25–27]. They work as feature extractors by means of convolution. The input is convolved with one or more kernels and the new representation is used to predict which class the input belongs to. Therefore, after training, the kernels generate a representation of the original input that is more suitable for discriminating samples assigned to different classes. Recently, CNNs have been applied to MRS(I) data to estimate tissue concentration of metabolites [28–30], to enhance spatial resolution of MRSI [31] and to asses MRSI spectral quality and filter artifacts [32, 33], but they have not yet been used in the classification of disease by MRSI. A challenge in the development of classification models with MRSI data is the limited number of cases available. MRSI data is in general scarce, preventing the use of deep neural network architectures with many parameters to learn. Therefore, in this paper we investigate the use of shallow CNN’s with only one hidden layer for classification of disease by MRSI.

Even though spectra and images are different data types, they are both characterized by feature locality: spatially for images and spectroscopic for spectra [34]. Feature locality means that values of neighboring features are highly correlated. These neighboring features are adjacent frequencies for spectra and nearby pixels for images. In this paper, we present a CNN method based on [35] to classify brain voxels exploiting such feature locality. To achieve this, we designed a type fusion approach, where spectroscopic and image data are jointly used to train a CNN with a single hidden convolutional layer that accounts for spectral locality for spectra and spatial locality for the images. Furthermore, we added a regularization term to the loss function to penalize large variations in weight values to avoid overfitting. Specifically, we considered a CNN with two input branches, each with a single hidden convolutional layer, in order to process both spectroscopic and image features of a brain voxel at the same time. Each branch consists of a single hidden convolutional layer. The output of these two branches is then merged and used as input to a prediction layer which outputs class predictions for the given brain voxel.

In this study, we applied our CNN method to ^1^H MRSI data from patients with brain tumors of different grades and to ^31^P MRSI data of Alzheimer’s disease patients and age-matched controls with the aim to classify tumor grades and to distinguish Alzheimer’s disease from healthy controls. This relatively simple CNN architecture is used to match the limited number of cases in the datasets and to facilitate the interpretability of the results in the form of relevant spectral regions.

We compared the Matthews correlation coefficient (MCC) score of the classification by CNN against classification with three other popular machine learning (ML) methods: Support Vector Machines with radial basis function kernel (SVM) [36], and two variations of Partial Least Squares Discriminant Analysis (PLS-DA) [37–40]. For further validation and interpretation of our model, we identified regions of the spectrum that are most informative for the classification as identified by the trained model. For this purpose we used stability selection [41] on the output of the hidden layer of the trained model to select relevant features. Then, we used deconvolution to map these selected features back into regions of the original spectrum. We evaluated whether the spectral regions mostly contributing to the classification are of clinical importance and whether the addition of MR images to the spectra improves the classification MCC score.

## Materials and methods

### Datasets

In this study, a brain tumor dataset [3] and an Alzheimer’s disease dataset [42] with both MR spectroscopic image and MR image data were explored. A summary of these datasets is presented in Table 1.

**Table 1:** Description of the MRSI datasets that have been used in our analysis. Metabolites peaks are quantified from MR spectra and these peaks correspond to brain metabolites. Spectra refer to MR spectra. Images refer to MR images.

Summary of MRSI Datasets	brain tumor	Alzheimer’s disease
# Healthy Subjects	4	31
# Patient Subjects	25	31
# Voxels	669	248
# ROIs	1	4
# Tissue classes	6	2
Data types	Metabolite Peaks/Spectra/Images	Spectra/Images

**Fig 1:**
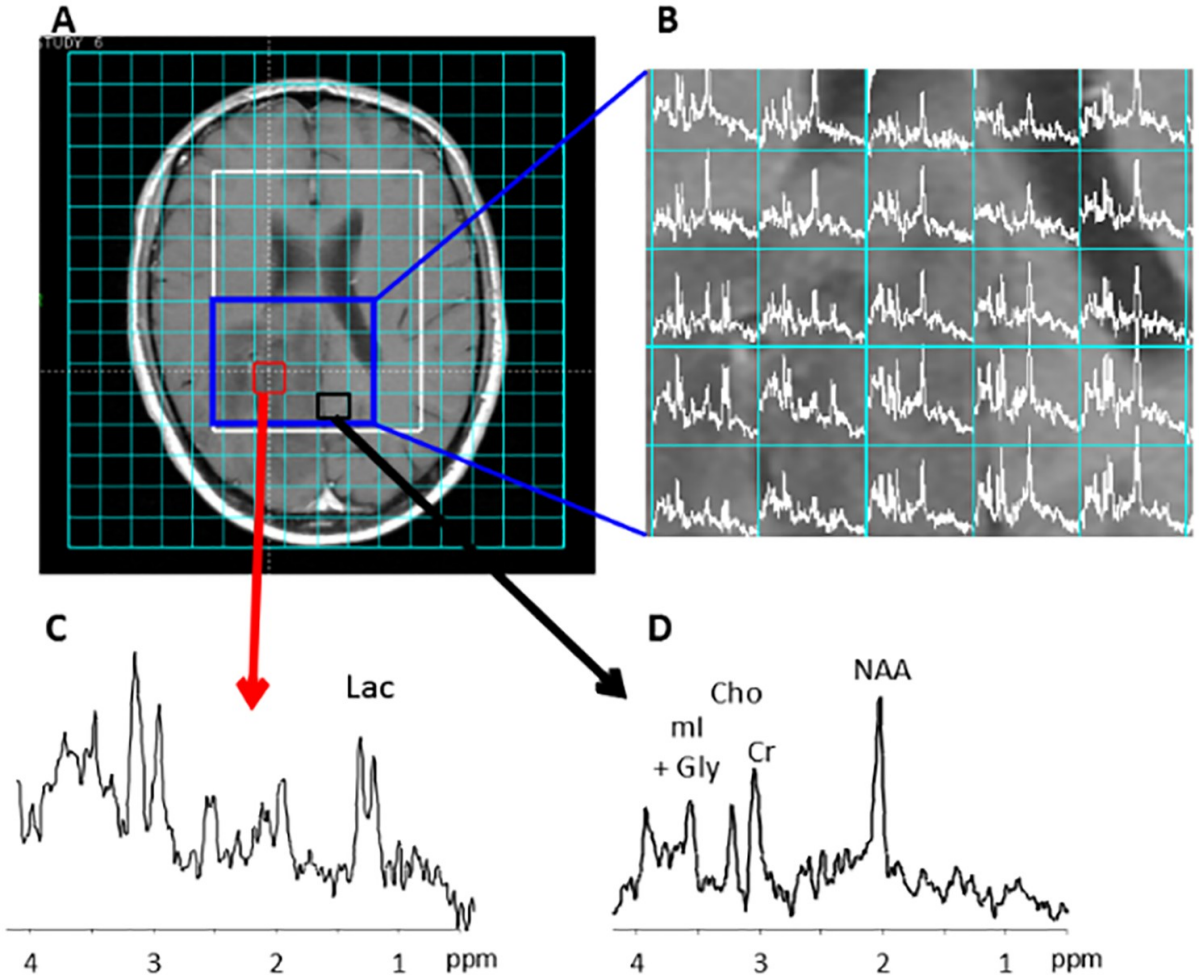
^1^*H* MRSI of the brain of a patient with a low grade glioma. (A) Post gadolinium T1 weighted MRI. The tumor appears as low intensity signal on the image. The ^1^*H* MRSI acquisition grid is shown on top of the image with the selected region of interest (white box). (B) Map of ^1^*H* MR spectra from selected box (blue) covering the tumorous area. (C) ^1^*H* MR spectrum of voxel in tumor. (D) ^1^*H* MR spectrum of voxel from an apparently benign brain area. The resonances of the methyl protons of lactate (Lac); N-aspartylaspartate (NAA); creatine (Cr) and choline (Cho) are indicated together with those of protons of myo-inositol (mI) and glycine (Gly).

The brain tumor dataset consists of 25 patients and 4 volunteers and was acquired under the INTERPRET protocol and quality control procedure [7, 43]. Two-dimensional ^1^*H* MR spectroscopic images (MRSI) were obtained at 1.5 T of a single slice of 12.5–15 mm covering the tumor area with a field of view (FOV) of 200 mm, a matrix size of 16 × 16, zero filled to 32 × 32 and STEAM region of interest (ROI) selection with a TE of 20 ms. Four different MR images (i.e. T1 Fig 1, T2, Pd and Gd) were used for the brain tumor dataset.

Postprocessing of the MR data was performed as described in [3, 4, 13]. Voxels were selected for six tissue classes as described in [3, 4]. Three classes correspond to the grade of the glial tumor (Grade II to grade IV), one corresponds to meningiomas, one to cerebral spinal fluid and one to normal tissue. The dataset contains on average 27.25 voxels per subject, but patients with grade II/III/IV tumor had fewer voxels per subject: respectively 17.60, 13.80 and 10. Apart from whole spectra also metabolite peak integrals, as extracted from these spectra in [13], were used in this study.

The Alzheimer’s disease dataset consists of 62 subjects, of which half were diagnosed with mild Alzheimer’s disease (n = 31, mean age 73.4 years, 13 men) and half were healthy control subjects, age and gender-matched to the Alzheimer’s disease group. Three dimensional ^31^P MRSI of the whole brain was obtained at 3 T with a nominal voxel size of 17.5 mm^3^. T1 weighted anatomical MR images were obtained at 1 mm isotropic voxels resolution.

For each subject, four ROIs were selected from the MRSI dataset, (each comprising a single voxel centered on the retrosplenial cortex (RSC), the anterior cingulate cortex (ACC), and the left and right hippocampus (HL and HR). Therefore, we have five different datasets, one for each brain area and a multi-region dataset consisting of all the four regions combined together as if they were different channels. After Fourier transformation of the raw MR data into the frequency domain, zero filling, apodization with a Gaussian filter and phase correction were applied. All post-processing was done in Matlab using MRS_MRI_libs libraries in which phase correction was partly automated.

For both datasets, full spectra, quantified peaks and images corresponding to each voxel have been considered for evaluating classification performances of our method and existing methods used for comparison.

### Convolutional neural networks

A convolutional neural network (CNN) uses hidden layers in which a convolution between their input and a set of *K* kernels of a certain size *N* is performed. After each convolutional layer, pooling layers can be used to progressively reduce the spatial size of the representation to reduce the number of parameters and computation in the network, and hence to also control overfitting. It is worth mentioning a few important hyper-parameters of CNNs:

- the size *N* of a kernel defines the range of action of the convolution and depends on the distribution of neighboring resonance frequencies;
- the stride *s* defines the step of the convolution when shifting the kernel over the whole input;
- the learning rate *η* determines how fast the optimization should converge to a local optimum;
- the regularization terms λ_1_ and λ_2_ determine the amount of regularization applied to the objective function optimized for reaching a local optimum.

*N* and *s* are related to convolution performed in the convolutional layer. *η*, λ_1_ and λ_2_ are parameters used for the whole CNN.

CNNs can use the structure of neighboring resonance frequencies to build a feature representation. This can be obtained by training a CNN to solve a prediction task. Thus, this new representation contains features that can lead to better predictions.

**Fig 2:**
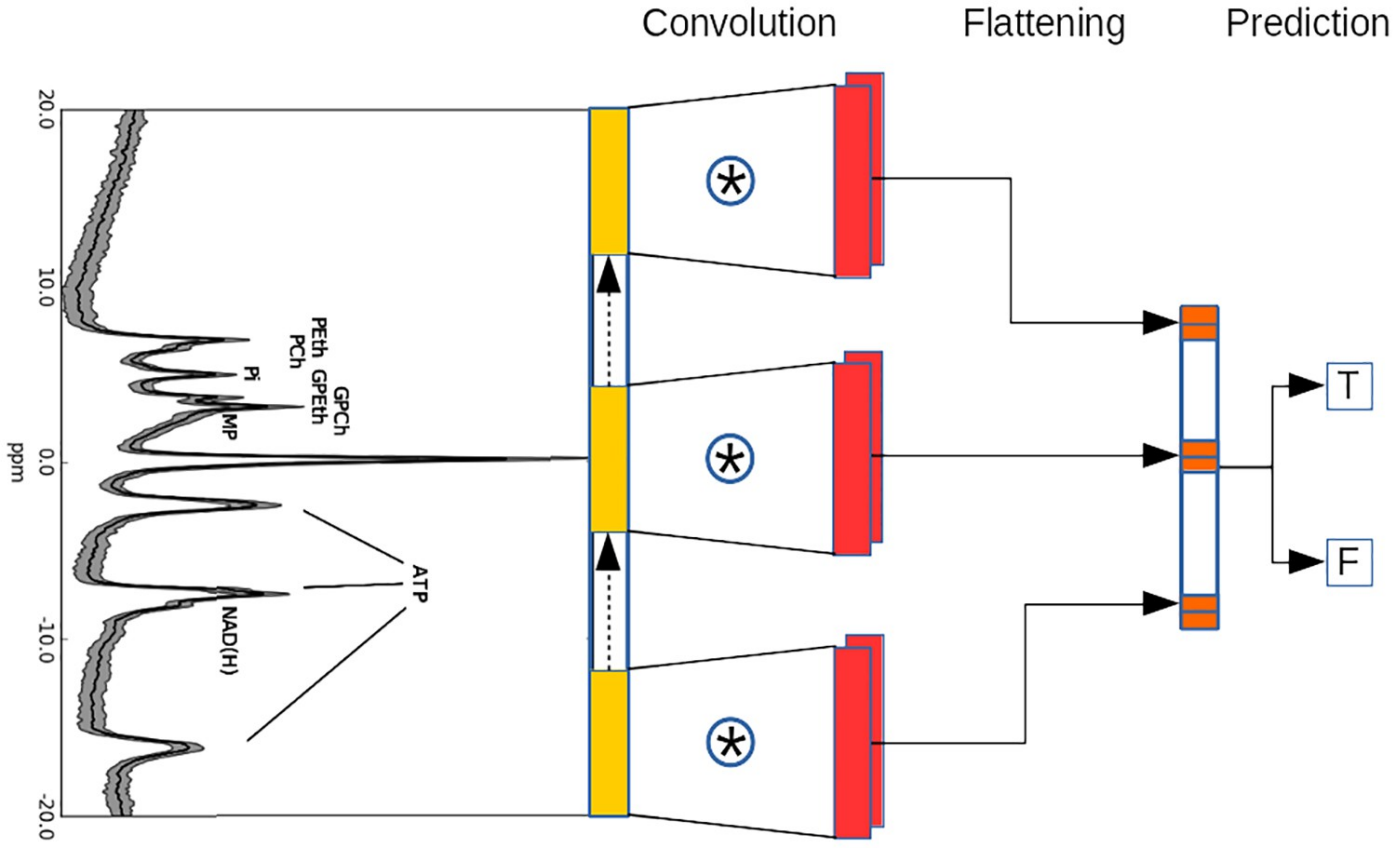
SL-CNN architecture and functioning for a 2 classes classification task (T or F to separate Alzheimer’s disease and normals). The convolution is performed by sliding each kernel of the convolutional layer over the input spectrum of the dataset. At every location (yellow), a vector multiplication is performed and sums the result onto the feature map (orange). Then, the output of the convolutional layer is reshaped into a vector (flattening) and used for prediction (T or F). In this example, 2 kernels (red) are applied.

We applied a single-layer Convolutional Neural Network (SL-CNN) architecture that we introduced for vibrational spectroscopic data classification [35]. A SL-CNN has a single hidden convolutional layer and uses an extra custom regularization term which penalizes large variations of values of neighboring kernels weights. Its architecture and functioning are shown in Fig 2. The architecture of SL-CNN allows it to tackle small and class-unbalanced datasets for which deeper CNNs would be likely to overfit on the most common class.

#### SL-CNN for multiple data types

**Fig 3:**
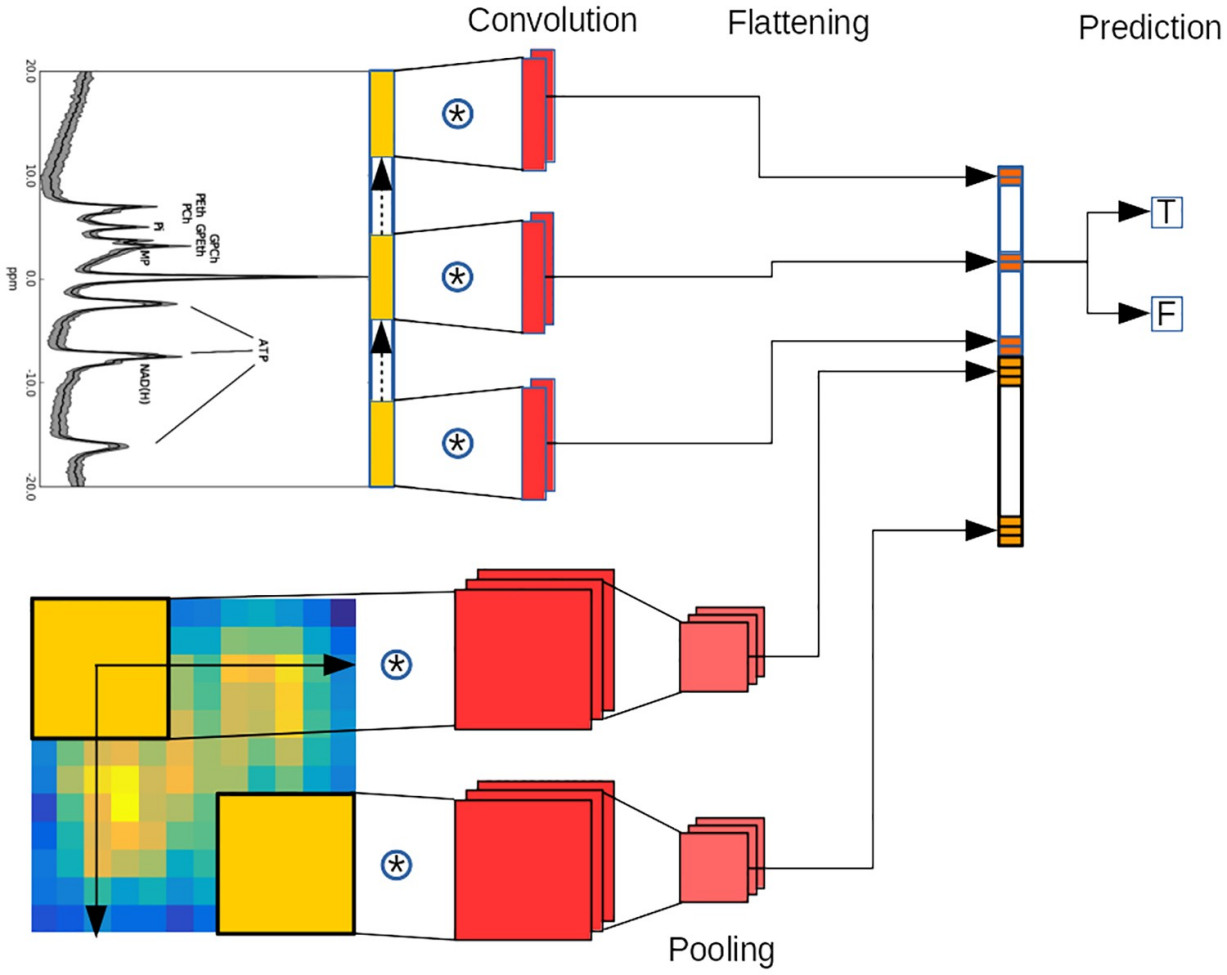
SL-CNN architecture and functioning for spectra and images for a 2 classes classification task (T or F to separate Alzheimer’s disease and normals). The convolution is performed by sliding each kernel of the convolutional layer over the input spectrum and the corresponding image. At every location (yellow), a vector or matrix multiplication for spectra or images respectively is performed and sums the result onto the feature map (orange). Then, a pooling layer is used to reduce the dimensionality of the convolutional layer output for images. Finally, the outputs from the different network branches are reshaped into a vector (flattening), merged and used for prediction (T or F). In this example, 2 kernels (red) are applied for spectra and 3 kernels (red) for images.

The very flexible architecture of artificial neural networks allows a design of various architectures for different data types. We integrated both spectra and images by merging different networks for image and spectral data. The SL-CNN architecture which works with spectra as inputs (see Fig 2) is modified by adding another branch that evaluates the spatial distribution of signal intensity in T1, T2, PD and Gd images (see Fig 3). The new branch of the neural network has a 2D convolutional and a pooling layer whose output is merged to the output of the other branch of the network. An MRI image and spectrum belonging to the same brain voxel are presented simultaneously to the two branches of the network. Next, the merged features are used as input for the last layer of the network, which outputs a classification.

### Identifying relevant spectral regions

An important part of the assessment and interpretation of a model is to find out which input features mostly determine its classification decision. Particular compounds (identifiable in spectra) are associated with a certain disease progression and we would like to determine whether this is also true for our model. On the other side, our analysis might identify compounds to be of importance for certain disease conditions, which were previously unknown to be involved.

In order to identify important regions in the original spectra we applied stability feature selection [41]. In problems where the number of features is much larger than the number of samples, which is the case for most MR spectroscopic datasets, stability selection is particularly effective.

Stability feature selection works as follows: feature selection is applied many times to different randomly selected subsets of the data (in our case we apply it to the convolutional layer output of the trained CNN). This feature selection is performed by retraining the last layer of CNN which can be seen as a logistic regression network having as input the output of the convolutional layer in response to different subsets of spectra, and as output the class prediction. After each retraining, features with positive coefficients are selected. The results over the subsets are then merged by considering for each feature the fraction of times it was selected. The features with the highest scores are considered to be the important ones [41].

**Fig 4:**
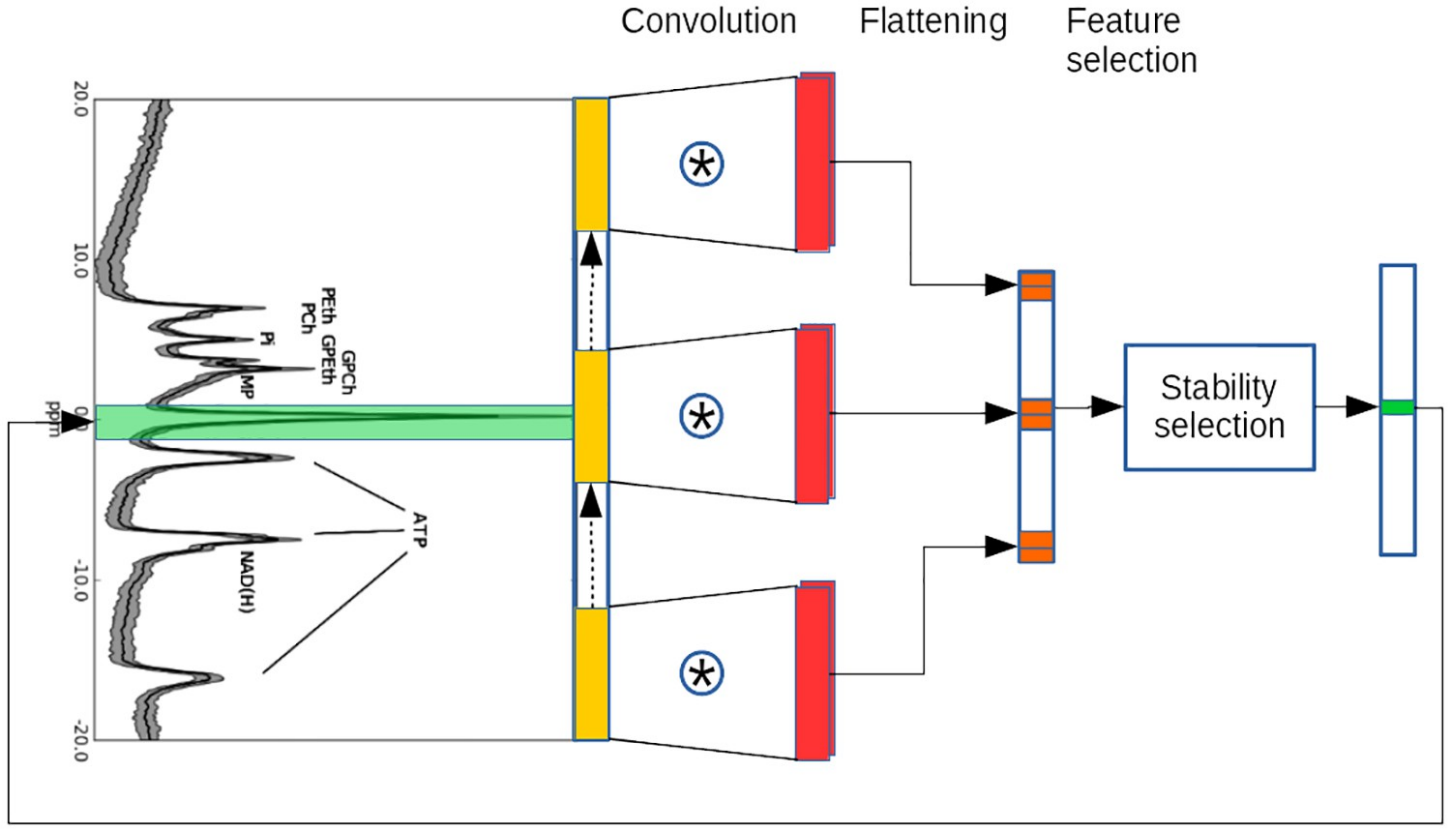
Example of identification of important spectral regions using stability selection on the output of SL-CNN. Stability selection is applied to the output of the convolutional layer after the neural network has been trained. Based on the convolutional layer parameters (i.e. number and size of the kernels and stride of the convolution), it is possible to map them to a specific region of the input spectrum.

After applying stability selection, we can unequivocally map the selected features of the convolutional layer output back to regions of the original input spectrum as schematically shown in Fig 4. In fact, each feature of the convolutional layer output has been obtained using a kernel of a certain size applied to a certain region of the original input spectrum, thus it is possible to find out which region of the original input spectrum contributed to each feature of the convolutional layer output.

### Other machine learning methods applied for comparison with SL-CNN

Support vector machines (SVMs) are applied in a large variety of classification problems because they are easy to use and commonly achieve very good results [44–46]. SVMs use the kernel trick to map the original data into a higher dimensional feature space in order to be able to separate samples belonging to different classes. Various non-linear functions can be used to specify the kernel. For this work, we used a common kernel function, the radial basis function (RBF). The kernel has a parameter *γ* that determines how far the influence of a single training example reaches. This kernel parameter is optimized along with the SVM regularization parameter C. Partial least squares analysis PLS-DA is a variant of Partial Least Squares PLS that uses discriminant analysis to perform classification. PLS is a regression method that aims to find a model by projecting the predicted and observed variables into a latent space [37, 47]. N-dimensional partial least squares discriminant analysis NPLS-DA is a generalization of PLS-DA for tensor data [38]. In a tensor dataset, data are available along multiple dimensions. For example in the Alzheimer’s disease dataset, for each subject, there are four spectra corresponding the four different regions of the brain. Therefore, each subject is represented by a vector of four spectra and the whole dataset is a rank 3 tensor (samples, wavelengths, regions). Using PLS-DA would require reshaping this rank 3 tensor to a matrix (samples, wavelengths of the four regions), while with NPLS-DA the tensor can be used directly.

Kernel partial least squares discriminant analysis KPLS-DA, like SVM, uses the kernel trick to transform the original data [40]. The resulting non-linear mapping should provide a representation of the data with improved capability to discriminate the classes. In this paper, we use the RBF kernel.

A crucial step in KPLS-DA is the optimization of the kernel which consists of selecting the best kernel hyper-parameters based on the MCC score of the resulting method.

KPLS-DA can also work with multiple views. An interesting advantage of KPLS-DA over NPLS-DA is that different views can be analyzed independently and then the resulting optimized kernel can be merged using a weighted average into a unique kernel as follows:

$$K=\sum_{i}^{M}a_{i}K_{i},$$

where *M* is the number of kernels to merge, and *a*_*i*_ is the weight associated with kernel *K*_*i*_. These weights *a*_*i*_ are tuned using the L1 norm $\sum_{i}^{M}{∥a_{i}∥}_{1}=1$ [48]. Multi-block partial least squares discriminant analysis MBPLS-DA is a version of PLS-DA that handles different data types in a non-trivial way (i.e. by reshaping and then concatenating features of different types) [39]. MBPLS-DA weighs different types for improving classification over the classical PLS-DA. This allows it to penalize or favor types that otherwise would give the same contribution for classification which can be disadvantageous when some data types are less informative than the rest.

MBPLS-DA is a widely used technique in the field of chemometrics for the purpose of exploring and modeling the relationships between several datasets to be predicted from several other datasets. In the case of only one data type available, MBPLS-DA is equivalent to PLS-DA.

### Validation protocol

For both datasets, we accounted for the potential classification bias that could result from using voxels of the same patient in the training and validation set [13]. In fact, voxels of a patient have similarities that are related to the specific patient and not ascribable to the whole group involved in the classifications. Specifically, we use leave-one-patient-out with two stages of nested cross-validation (LOPO-CV). Nested cross-validation has become a common and recommended a method of choice for algorithm comparisons for small to moderately-sized datasets [49]. Leave one out cross validation is a widely used technique to assess classification especially with small datasets [50–53]. The inner cross-validation is used to find the best combination of hyper-parameters values. To this aim, multiple runs of cross-validation are repeated with different hyper-parameters values. The outer cross-validation then uses the best hyper-parameters to asses the performances of the methods, in terms of MCC. The coefficient provides a balanced measure which can be used even if the classes are of very different sizes [54].

Given that LOPO-CV procedure maximizes test-set variance and does not yield stable estimates of predictive accuracy, we also considered 3-fold cross validation on the best performing method for each dataset and reported the resulting MCC.

## Results

We used single spectroscopic imaging and MR imaging data and their combination to compare the SL-CNN method with the other machine learning methods. The data were rescaled in a range between [0, 1] before being used. For SVM we concatenated images and spectra into a unique sample.

Depending on the number of types and data views, we employed different versions of PLS-DA:

- PLS-DA, for single type, single view data;
- NPLS-DA, for single type, multiple views data;
- MBPLS-DA, for multiple types.

Only MBPLS-DA can handle both multiple types and multiple views and treat data views as different types. We refer to all these methods as PLS-DA in the tables and specify which variant was used if needed.

We also compared our SL-CNN results on brain tumor typing with results published previously, in which quadratic discriminant analysis was used on metabolite peaks and average signal intensity of MRI T1, T2, PD and Gd images of the same voxel location [13]. For a proper comparison we had to submit the results of this analysis to the same LOPO-CV evaluation procedure as used in the present study.

In order to tune the hyper-parameters of the models (see Supporting information Section ‘Hyper-parameters’), we used the Random Grid Search Cross-Validation framework (RGS-CV) [55]. The optimal hyper-parameter values we found for the SL-CNN algorithm applied to MR data of patients with a brain tumor or Alzheimer’s disease are presented in Table S1 in S1 File. As can be seen, the ranges of optimal values do not substantially differ between the considered datasets except for the regularization parameters λ_1_ and λ_2_ which are intrinsically dependent on the type of dataset.

### Brain tumor dataset

**Fig 5:**
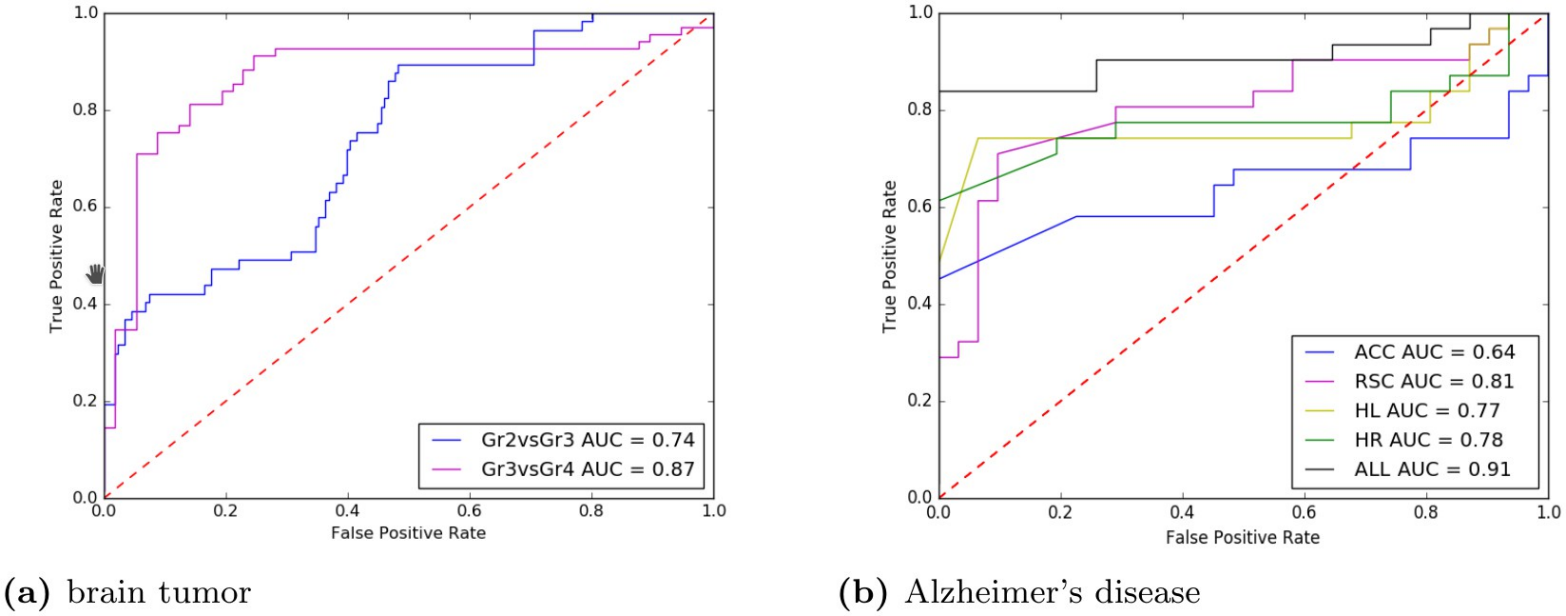
ROC curves corresponding to the best SL-CNN’s classification models. (a) corresponds to the separation of brain glioma tumor grades and (b) to Alzheimer’s disease from normal brain. The highest AUC for the separation of brain tumor grades was achieved for grade 3 versus grade 4. The highest AUC to identify Alzheimer’s disease was achieved for a combination of all 4 investigated areas. AUC is the area under the ROC curve. AUC measures how true positive rate and false positive rate trade off.

A T1 weighted MR image and ^1^*H* MRSI data of the brain of a patient with a low grade oligodendroglioma are shown in Fig 1. ^1^*H* MR spectra of voxels from the MRSI recording of that tumor and resonance peaks for various brain metabolites can be observed such as the methylprotons of choline, creatine, N-acetylaspartate and lactate (see also Fig 1C and 1D). In tumors typically the N-acetylaspartate signal is decreased and the choline signal increased, whereas a resonance for lactate may appear. The spectral region close to lactate also contains resonances for lipids and alanine, which may also increase in tumors [1]. The MRI and MRSI information sampled from multiple glioma patients for a complete brain tumor dataset was used to assess two challenging classification tasks: between gliomas with grade II and grade III (Table 2) and between gliomas with grade III and grade IV (Table 3). The results demonstrate that for each type of data or combination of data the SL-CNN MCC score to discriminate between these grades was comparable to or better than that of the other existing methods. In particular the SL-CNN result of the combination of the full MR spectra and signal intensity T1, T2, PD and Gd MRI data was superior to that of the other methods. The SL-CNN accuracies of this combination and its ROC analysis (Fig 5a) reveals that the SL-CNN applied to this data performs best for the discriminative classification of the glioma grades III and IV with an AUC of 0.87 as compared to 0.74 for the discrimination of grades II and III.

**Table 2:** Average classification MCC score assessed by LOPO-CV to separate voxels with grade II versus grade III tissue in the brain tumor dataset. MBPLS-DA is used instead of PLS-DA for the last line given the multiple types (i.e. MR spectra and MR images). Average MRI data are obtained from MR images by averaging pixels intensities for all voxels. Metabolites peaks, corresponding to brain metabolites, are quantified from MR spectra. Full MR spectra means that complete spectra are used as input data. Standard deviation is reported below each score between brackets. The best results are underlined.

Input Data	SL-CNN	SVM	PLS-DA	KPLS-DA
Metabolite peaks and average MRI data	0.3697 (0.0078)	0.2968 (0.0048)	0.2968 (0.0088)	0.3697 (0.0079)
MR spectra	0.5822 (0.0017)	0.4096 (0.0075)	0.2432 (0.001)^(N)^	0.3882 (0.0069)
MR images	0.3697 (0.0046)	0.3697 (0.0067)	0.1903 (0.0078)	0.2968 (0.0036)
MR spectra and MR images	0.6657 (0.0078)	0.3697 (0.0008)	0.2434 (0.0045)^(MB)^	0.4636 (0.0068)

**Table 3:** Average classification MCC score assessed by LOPO-CV to separate voxels with the grade III versus grade IV in the brain tumor dataset. MBPLS-DA is used instead of PLS-DA for the last line given the multiple types (i.e. MR spectra and MR images). Average MRI data are obtained from MR images by averaging pixels intensities for all voxels. Metabolites peaks, corresponding to brain metabolites, are quantified from MR spectra. Full MR spectra means that complete MR spectra are used as input data. Standard deviation is reported below each score between brackets. The best results are underlined.

Input Data	SL-CNN	SVM	PLS-DA	KPLS-DA
Metabolite peaks and average MRI data	0.6600 (0.0077)	0.6580 (0.0079)	0.5180 (0.0033)	0.6580 (0.0004)
MR spectra	0.7382 (0.0014)	0.6580 (0.009)	0.4381 (0.0021)^(N)^	0.6580 (0.0043)
MR images	0.6580 (0.002)	0.6561 (0.0018)	0.3384 (0.0017)	0.4381 (0.004)
MR spectra and images	0.8387 (0.0093)	0.7985 (0.0028)	0.4381 (0.0066)^(MB)^	0.6981 (0.0053)

A quadratic discriminant classification analysis of the same glioma dataset [13], but using the same LOPO-CV evaluation as applied in the current paper, resulted in 0.3659 MCC score in separating grade II and grade III and 0.1445 MCC score in separating grade III and grade IV, which is significantly worse than our results using SL-CNN.

The MCC score obtained using 3 fold cross-validation protocol for SL-CNN with MR spectra and images as input is 0.7151 for the classification of grade II and grade III and 0.8822 for the classification of grade III and grade IV.

**Fig 6:**
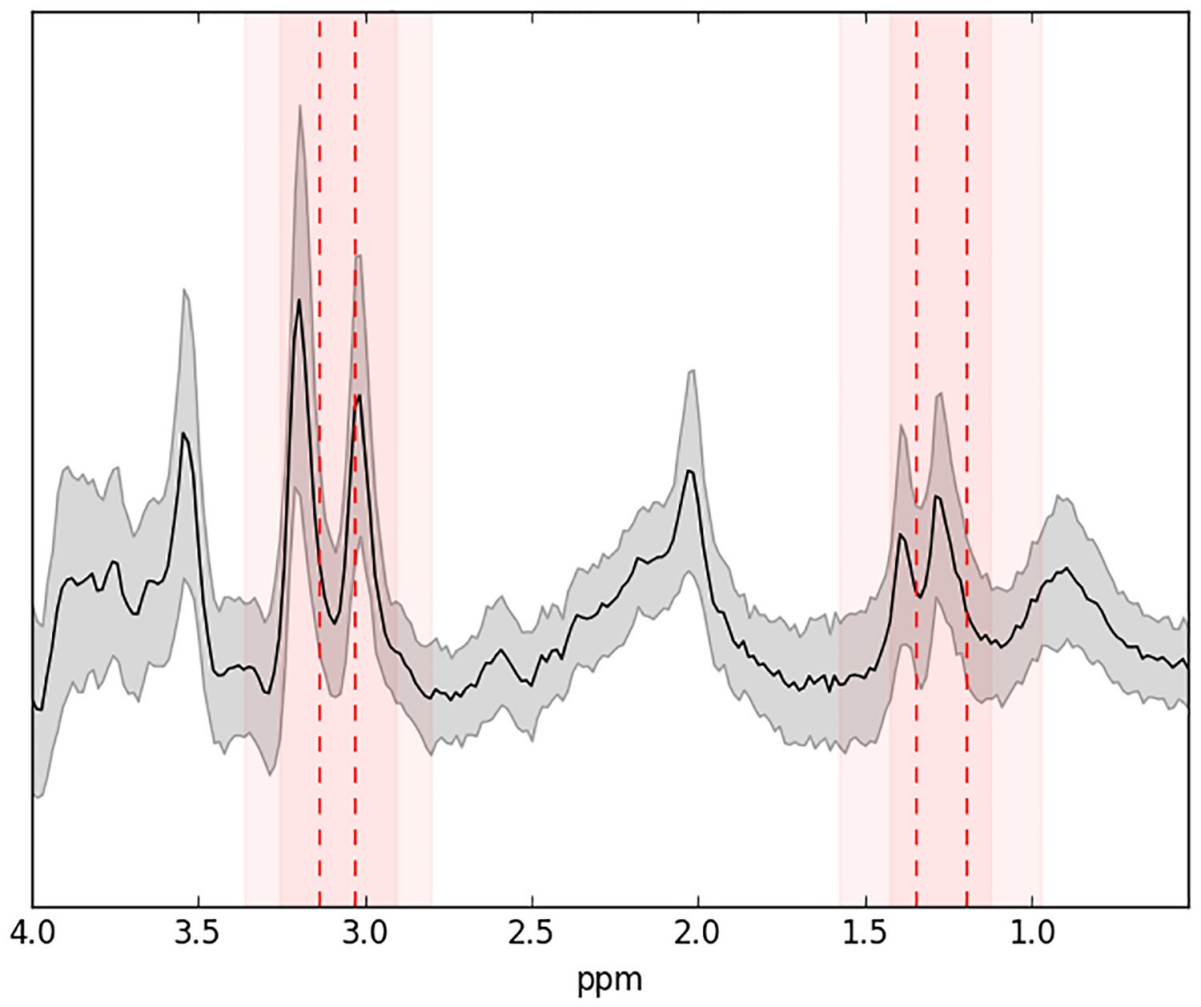
Important spectral regions for the separation of brain tumor dataset grade II vs grade III. The average spectrum for each area is plotted as a reference in (solid) black and the gray zone around it shows the standard deviation. The red dotted lines indicate the center of the most important regions (found by stability selection) and the red shaded areas around them indicate their size. These areas were obtained using stability feature selection on the output of the convolutional layer of the trained SL-CNN using only spectra as inputs.

The stability feature selection applied to the output of the SL-CNN convolutional layer identified the spectral region at the lactate and lipid signals and a region at the choline and creatine signals as contributing most to the discrimination between of grade II and III (Fig 6). The highlighted spectral areas were selected using a very conservative selection threshold (0.96) as specified in [41].

### Alzheimer’s disease dataset

**Fig 7:**
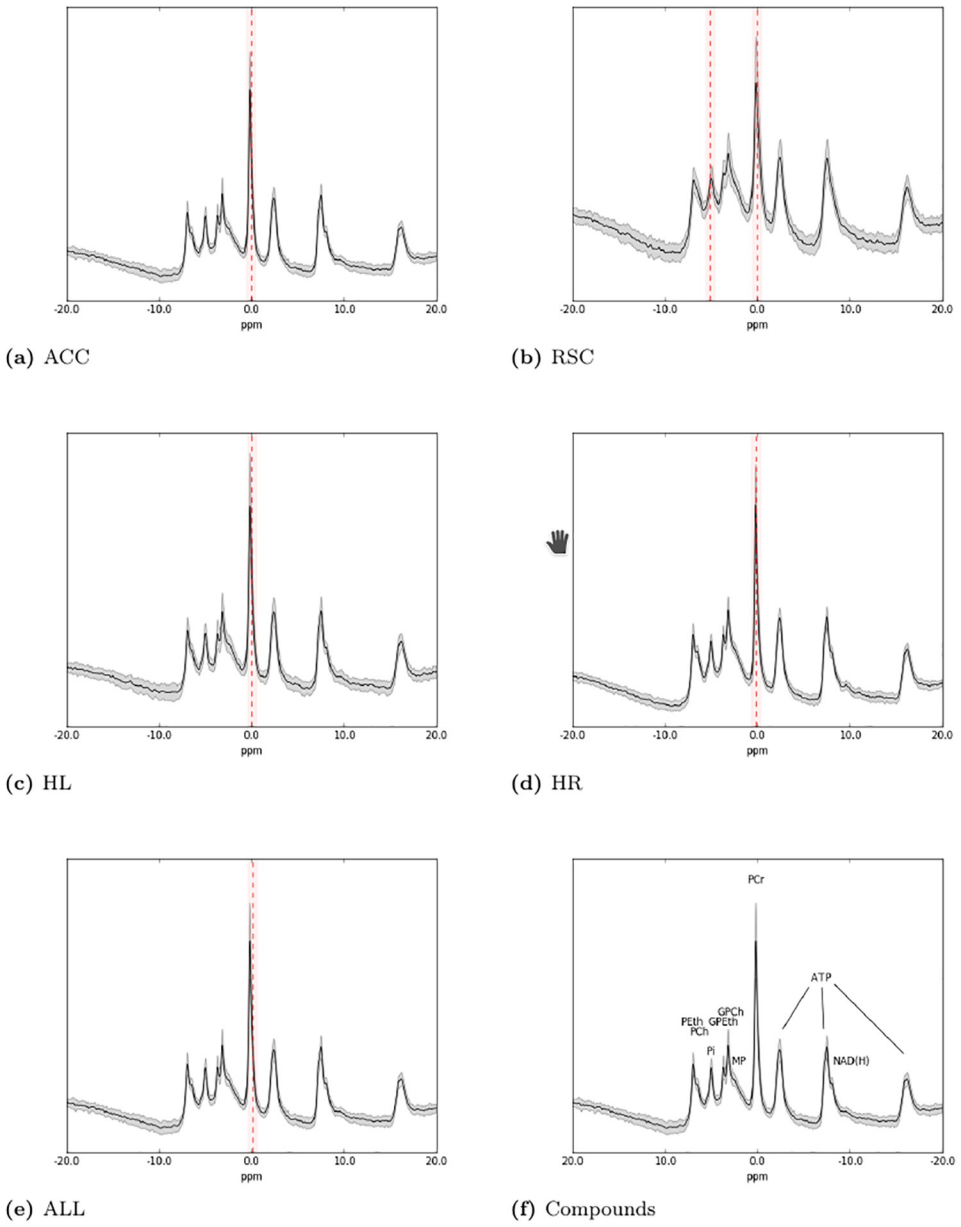
^31^*P* MR spectra of the brain of patients with Alzheimer’s disease. Important spectral regions identified for the Alzheimer’s disease dataset are shown for the ACC (a), the RSC (b), the HL (c), the HR (d) and their combination (e). A reference spectrum with annotated peaks is shown in (f). The average spectrum for each area is plotted as a reference in (solid) black and the gray zone around it shows the standard deviation.

The binary task of discriminating Alzheimer’s disease patients from healthy age-matched subjects was addressed by considering ^31^*P* MR spectra of single voxels for each of four selected brain regions of interest (ROIs). Therefore, we have five datasets, one for each brain ROI and a multiple ROI dataset built by merging these four datasets. An example of ^31^*P* MR spectra of multiple regions, showing resonances for various phosphorylated metabolites indicated, is presented in Fig 7. The results of the machine learning analysis demonstrate (see Table 4) that the SL-CNN method is superior in the classification of Alzheimer’s disease compared to the other methods for each ROI, each type of data and for the combination of data. The best MCC score (0.7156) was achieved for the combination of MR spectra and images of all four ROIs together. The MCC score using the T1 weighted MR image data was the least and this data hardly contributed to the MCC score of detecting Alzheimer’s disease for any data type combination. An ROC analysis of the discrimination between Alzheimer’s disease and healthy persons resulted in an AUC of 0.91 using the combination of all four areas. ROC AUC curve of combined brain areas is statistically significantly different (i.e. p-value <0.05) with respect to AUC ROC curves of single areas according to the DeLong’s algorithm for comparing AUC ROC curves [56].

**Table 4:** Average classification MCC score assessed by LOPO-CV to separate Alzheimer’s disease patients (31) from healthy controls (31). (N) steads for NPLS-DA (PLS-DA for tensor data), (MB) steads for MBPLS-DA (PLS-DA for data with multiple types). Standard deviation is reported below each score between brackets. The best results are underlined.

Input Data	Brain Areas	SL-CNN	SVM	PLS-DA	KPLS-DA
MR spectra	ACC	0.4610 (0.0039)	0.2113 (0.0086)	0.1000 (0.0014)	0.3592 (0.0072)
	RSC	0.6247 (0.0047)	0.4600 (0.0036)	0.4800 (0.0075)	0.5400 (0.0039)
	HL	0.5649 (0.0048)	0.1200 (0.0065)	0.1268 (0.0053)	0.1600 (0.001)
	HR	0.6647 (0.0036)	0.2200 (0.0085)	0.0600 (0.0085)	0.2200 (0.005)
	ALL	0.7096 (0.0018)	0.4712 (0.0012)	0.4780 (0.0083)^(N)^	0.5303 (0.0013)
MR Images	ACC	0.3000 (0.0064)	0.0423 (0.0052)	0.0423 (0.0001)	0.1000 (0.0042)
	RSC	0.3400 (0.0097)	0.0400 (0.0052)	0.0423 (0.0099)	0.1056 (0.0035)
	HL	0.2336 (0.0099)	0.0400 (0.0008)	0.0400 (0.0057)	0.0423 (0.0051)
	HR	0.2400 (0.0067)	0.0423 (0.0047)	0.0423 (0.0026)	0.1056 (0.0054)
	ALL	0.2336 (0.0021)	0.0400 (0.0021)	0.0423 (0.0091)^(N)^	0.1056 (0.0071)
MR spectra and images	ACC	0.4689 (0.0081)	0.2241 (0.0018)	0.4451 (0.0071)^(MB)^	0.4597 (0.0003)
	RSC	0.6319 (0.0006)	0.4711 (0.0087)	0.4800 (0.0041)^(MB)^	0.5521 (0.0006)
	HL	0.5649 (0.009)	0.1423 (0.0031)	0.2829 (0.0081)^(MB)^	0.3976 (0.0034)
	HR	0.6724 (0.0039)	0.2400 (0.009)	0.4869 (0.0001)^(MB)^	0.4992 (0.0055)
	ALL	0.7156 (0.0066)	0.4712 (0.0025)	0.4376 (0.0064)^(MB)^	0.5500 (0.0086)

The MCC scores obtained using 3 fold cross-validation protocol for SL-CNN with MR spectra and images as input of all the brain areas is 0.7589 for the classification of Alzheimer’s disease patients from healthy age-matched subjects.

Stability selection applied to the output of the convolutional layer, again using a very conservative selection threshold of 0.96, revealed that the spectral regions with the phosphocreatine (PCr) and inorganic phosphate (Pi) peaks are the most important contributions to the classification of Alzheimer’s disease and healthy persons (Fig 7).

Although our previous study on this Alzheimer’s disease dataset did not report classification MCC score values [42], we estimated this MCC score by using the PCr peak values reported in this study, as these showed a statistically significant difference between healthy and Alzheimer’s disease subjects. Assuming that the reported PCr peak values are normally distributed and using the mean and standard deviation values for each brain area we estimate MCC score of 0.0528 for ACC, 0.3185 for RSC, 0.3447 for HL, 0.4125 for HR and 0.0954 for their combination. These classification MCC scores are far below for what we achieved with the SL-CNN method in the present study Table 4.

## Discussion

In this paper we present a new SL-CNN algorithm to analyze MRSI data and demonstrate that it can successfully be applied for diagnostic purposes in the classification of diseases in the human brain. The method, which is based on a CNN algorithm introduced to analyze vibrational spectroscopy data [35], can integrate spectral and image features. Commonly, in applications, CNNs have a complex structure with several layers (e.g U-Net [31]) requiring a lot of data to properly train the network. We developed a less complex CNN, facilitating that less data is required to train the network model. Specifically, this algorithm was able to classify glioma grades from ^1^*H* MRSI data and patients with Alzheimer’s disease from ^31^*P* MRSI data. The SL-CNN algorithm outperformed the application of other popular ML methods in its classification MCC score of both investigated datasets. There are several reasons why it is preferable to use convolutional layers for the analysis of MRSI data instead of artificial neural networks with fully connected layers. The main reason is that neighboring resonance frequencies of the spectra are correlated. In fact, it is reasonable to assume that values of neighboring resonance frequencies cannot differ substantially. The same principle applies to neighboring pixels in MRI. Therefore, we exploited spectral locality with convolutions while doing feature extraction facilitating interpretation of the developed model. Additionally, such a network structure has the advantage of a reduced set of network parameters that need to be trained from the data [35].

In general, the combined use of different MR data types (spectra and images) improves the performance of the SL-CNN method, which was also noted in the application of other ML methods [4]. Both for the brain tumor and Alzheimer’s disease datasets the information of the MR image in the voxels that were analyzed performed poorly compared to that of the MRS. Apparently the MR spectra contain information about metabolites that is more specific for the diseases examined here than the information in the rudimentary MR images used in this study. Obviously, the inclusion of data obtained by more advanced MRI methods, such as diffusion- and perfusion-weighted imaging in the case of the brain tumors [57, 58], may further improve the performance of the SL-CNN algorithm. In addition, in particular for the Alzheimer disease dataset, we only used image information from the selected voxels, instead of larger areas defined by anatomical (lesion) boundaries. In the case of the brain tumor dataset the heterogeneity of tumor lesions may have hampered proper classification of voxels. Although the tumors have been generally classified to a specific grade by consensus in the pathologist panel, including in most cases a biopsy report [43, 59], tumor lesion are often heterogenous. Assuming that the tumor voxels have been properly selected, avoiding CSF and healthy tissue contributions, it still may be that the identified lesions contain tissue with more than one tumor grade [60]. For instance, nosologic imaging of the current MRSI dataset indicated that lesions identified as grade II may contain grade III components and vice versa [9].

Furthermore, the limited number of subjects and the variable number of voxels per subject for some classes is expected to hamper the classification of tumor grades. For example, since there are limited grade III voxels/patient and only very few patients having grade III voxels, the nested cross-validation might influence negatively their correct classification.

Previous classification studies employing this brain tumor dataset did not account for similarities related to the specific patient and not ascribable to the whole group involved in the classifications. For example, multi-class classification using SVM was performed without applying LOPO-CV [17]. The chosen validation protocol (i.e. stratified random sampling) is expected to bias the classification results. Furthermore since the problem was approached by a multi-class classification, the chances for the trained model to overfit were higher. Because of the chosen validation protocol, the trained model will be biased by modeling similarities of voxels of the same patient rather than determined by characteristics of classes to improve classification MCC score.

In [13], the LOPO-CV procedure was not implemented correctly. This mostly affected the grade II vs grade III and grade III vs grade IV classification. We fixed the issue and computed the new classification results to be able to compare with SL-CNN classification MCC score. The resulting classification accuracies are: 68.3% and 57.8% for grade II vs grade III and grade III vs grade IV respectively, which are lower than the classification accuracies achieved with SL-CNN.

The stability selection of the SL-CNN method identified spectral regions that were important in the classifications. These regions are similar to those reported in previous studies of the same datasets of brain tumor POSTMA201187 and Alzheimer’s disease alzheimer dataset.

The highlighted important regions for the brain tumor dataset to discriminate grade II vs grade III correspond to the following spectroscopic ranges mentioned in [13]: 3.115–3.265 ppm (Cho), 2.955–3.105 ppm (Cr2) and 1.395–1.545 ppm (lactate + lipids + alanine). In a previous study of the same data, Cr2 and lactate + lipids + alanine were also important in the grade classification but not Cho [13]. However, differences in the intensity of the Cho signal have been consistently found to be important in the differentiation of grade II and grade III [58, 61]. The levels of the resonances of other compounds, such myo-inositol/glycine and NAA, msy also contribute to grade differentiation, but are less important.

The Alzheimer’s disease dataset consists of multiple brain areas. Thus, we used the single and combined data from all the areas to investigate which spectral regions are important according to stability selection. The main peak (PCr) located at 0.0 ppm was always highlighted as an important spectral region. This is in agreement with the analysis by [42] where PCr, a metabolite involved in brain energy metabolism, was found to be the main indicator of Alzheimer’s disease for all the brain areas except ACC. Thus, stability selection suggests that our method bases its predictions mostly on the PCr peak for all the brain areas and their combination. Only for the RSC brain area, stability selection highlights inorganic phosphate (Pi) (also involved in brain energy metabolism) as well as PCr. By including more brain regions and more advanced MRI data it is expected that the performance of the SL-CNN will be improved.

By looking at the average best hyper-parameter values identified using RGS-CV in Table S1 in S1 File, we can see that they are very similar for both the datasets. Only the stride value *s* is higher for the brain tumor dataset than for the Alzheimer’s disease dataset. This is probably due to a different distribution of important spectroscopic regions in MR spectra of Alzheimer’s disease compared to those of the brain tumors.

## Conclusion

In this work, we explored the use of convolutional neural networks for the classification of brain diseases using MRSI data, complemented by MRI data. We demonstrated the benefit of considering spectroscopic and image modalities together leading to an improved MCC score.

We validated and interpreted our models by investigating spectroscopic regions important in classification as identified by stability feature selection and by comparing the identified regions with those reported by others. Although a full proof of the diagnostic practicality of our new CNN approach requires inclusion of more data, it is encouraging that a simple CNN architecture with an ad-hoc loss function enabled to uncover regions important in the diagnosis of both brain tumor and Alzheimer’s disease, in agreement with previous findings reported in the literature.

Future work includes the use of more modalities. When available, such information could help to increase diagnostic performance, as shown by studies on Alzheimer’s disease notably ADNI, which utilizes data, including MRI and PET images, genetics, cognitive tests, CSF and blood biomarkers as predictors of the disease. Also, it might be interesting to investigate whether coupling the latent features with a classical radiomics approach can improve the classification performance.

## Supporting information

S1 File (PDF)
