# Supplementary material for: Convolutional neural networks to predict brain tumor grades and Alzheimer’s disease with MR spectroscopic imaging data
Source: PLoS One. 2022 Aug 24;17(8):e0268881. doi: 10.1371/journal.pone.0268881 (PMC9401174; doi:10.1371/journal.pone.0268881)
Supplement: S1 File — (PDF) [file pone.0268881.s001.pdf]

Convolutional neural networks to predict brain tumor grades and Alzheimer's disease with MR Spectroscopic Imaging data

Jacopo Acquarelli[1,2*], Twan van Laarhoven[1], Geert J. Postma[2], Jeroen J. Jansen[2], Anne Rijpma[3], Sjaak van Asten[3], Arend Heerschap[3], Lutgarde M.C. Buydens[2], Elena Marchiori[2*],

1 Radboud University Nijmegen, Institute for Computing and Information Science, Nijmegen, The Netherlands
2 Radboud University Nijmegen, Institute for Molecules and Materials, Nijmegen, The Netherlands
3 Radboud University Medical Center Nijmegen, Department of Radiology and Nuclear Medicine, Nijmegen, The Netherlands

* jacquarelli@cs.ru.nl, elenam@cs.ru.nl, Arend.Heerschap@radboudumc.nl

Hyper-parameters

The hyper-parameters of the SL-CNN model and their range of values (given between brackets), as used in our experiments are:

- number of kernels of the 1D convolutional layer: $\#kernels_{1D} \in \{4, 8, 16, 32\}$
- size of kernels of the 1D convolutional layer: $N_{1D} \in \{3, 91\}$
- stride of the 1D convolution: $s_{1D} \in \{1, 4\}$
- number of kernels of the 2D convolutional layer: $\#kernels_{2D} \in \{4, 8\}$
- size of kernels of the 2D convolutional layer: $N_{2D} \in \{3, 5, 7\}$
- stride of the 2D convolution: $s_{2D} \in \{1, 2\}$
- parameters in the regularization terms: $\lambda_1, \lambda_2 = 10^n$ where $n \in \{-4, 4\}$
- learning rate: $\eta = 10^n$ where $n \in \{-4, -1\}$

For SVM with RBF kernel we tuned the following terms:

- RBF parameter $\gamma \in 10^n$ where $n \in \{-4, 4\}$
- regularization constant $C \in 10^n$ where $n \in \{-4, 4\}$

For PLS-DA we used:

- number of latent components $\#components \in \{1, 20\}$
- different data rescaling methods (mean centering, auto-scaling and log scaling).

For KPLS-DA we used:

- number of latent components $\#components \in \{1, 20\}$
- RBF parameter $\gamma \in 10^n$ where $n \in \{-4, 4\}$
- kernels' weights a_i s.t. $\sum_i a_i K_i = 1$ in the case of multiple ways for the same sample (i.e. the 4 brain areas of Alzheimer's disease) or multiple types (i.e. spectra and images).

Tables

Parameters	brain tumor	Alzheimer's disease
$\#kernels_{1D}$	16 ± 11	14 ± 10
N_{1D}	35 ± 21	35 ± 19
s_{1D}	9 ± 6	3 ± 2
$\#kernels_{2D}$	4 ± 0	4 ± 1
N_{2D}	5 ± 1	5 ± 0
s_{2D}	2 ± 1	2 ± 0
λ_1	88 ± 255	120 ± 305
λ_2	3 ± 4	2 ± 4
η	0.10 ± 0.20	0.07 ± 0.16

Table S1. Average hyper-parameter values for SL-CNN selected using RGS-CV.
